# Integration Analysis of Bayesian and Machine Learning for Heterogeneity, Biomarkers, and Optimal Combination Regimens of Pucotenlimab in Solid Tumors

**DOI:** 10.1002/cam4.71893

**Published:** 2026-04-27

**Authors:** Yingge He, Changqing Gao, Shiyan Zhang, Yonghui Hao, Shuning He, Ke Peng, Liqi Li

**Affiliations:** ^1^ Department of Plastic and Cosmetic Surgery, Xinqiao Hospital Army Medical University Chongqing China; ^2^ Department of General Surgery, Xinqiao Hospital Army Medical University Chongqing China

**Keywords:** Bayesian meta‐analysis, immune‐related adverse events (irAEs), machine learning, network meta‐analysis, PD‐1 inhibitor, pucotenlimab (HX008), solid tumors

## Abstract

The efficacy of PD‐1 inhibitor pucotenlimab (HX008) in solid tumors exhibits heterogeneity. This study integrated data from 6 clinical trials (covering gastric/gastroesophageal junction cancer, triple‐negative breast cancer, melanoma, and dMMR/MSI‐H solid tumors) using Bayesian meta‐analysis, machine learning (optimal XGBoost AUC = 0.86), and network meta‐analysis to construct an integrated “efficacy‐prediction‐safety” framework. Bayesian analysis showed pucotenlimab significantly improved outcomes versus control (ORR OR = 4.82, 95% CrI: 3.65–6.38; PFS HR = 0.41, 0.32–0.52; OS HR = 0.37, 0.26–0.51). Subgroups revealed TNBC patients with gemcitabine/cisplatin achieved highest ORR (80.6%, 62.5%–92.6%), while mucosal melanoma showed lowest response (8.7%, 1.1%–28.0%). Combination therapy demonstrated superior efficacy to monotherapy (ORR OR: 5.91 vs. 2.35). Machine learning identified 4 efficacy biomarkers (KMT2D mutation, post‐treatment NLR decrease, PD‐L1 CPS ≥ 1, high eotaxin) and 3 irAE risk factors (baseline NLR ≥ 4, irinotecan combination, high VEGF). Network analysis recommended regimens: gemcitabine/cisplatin for TNBC (SUCRA = 95.7%), oxaliplatin/capecitabine for G/GEJ cancer (ORR = 60.0% vs. irinotecan 27.6%, HR = 0.45). The integrated model classified high‐benefit (≥ 3 points; ORR 78.2%) and low‐benefit (≤ 0 points; ORR 28.3%) groups, plus high‐risk (≤ −2 points; grade ≥ 3 irAEs 41.2%) and low‐risk (≥ 1 point; irAEs 3.5%) groups, validated by decision curve analysis. This defines precise application scenarios and provides an extensible analytical paradigm.

## 1 Introduction

### 1.1 Research Background

PD‐1 inhibitors have become the cornerstone of treatment for advanced solid tumors [1, 2, 3, 4, 5, 6, 7, 8, 9, 10], but significant efficacy differences exist among different tumor types, treatment regimens, and individual patients [8, 11, 12]. For example, the objective response rate (ORR) can range from 10% to 80% in gastric/gastroesophageal junction cancer [13, 14], triple‐negative breast cancer [15], and melanoma. Traditional frequentist meta‐analysis is difficult to accurately quantify the effect uncertainty of small‐sample studies (such as phase Ib/II trials). In small‐sample immunotherapy studies, random errors in efficacy and safety may lead to biased conclusions, thus requiring a more robust analytical framework [16, 17].

Pucotenlimab (HX008) is a humanized IgG4 anti‐PD‐1 monoclonal antibody engineered with Fc segment mutations (S228P/S254T/V308P/N434A) [18, 19, 20]. It has an extended half‐life and no antibody‐dependent cellular cytotoxicity (ADCC) or complement‐dependent cytotoxicity (CDC) activity [21, 22], which avoids killing immune cells expressing PD‐1 [5, 23]. Currently, six studies covering various tumor types include monotherapy and combination chemotherapy regimens [24, 25, 26, 27], but there is a lack of systematic integration analysis [1, 2, 3, 4, 5, 6]. In terms of efficacy, the combined effects and sources of heterogeneity of ORR, PFS, and OS across different tumor types have not been clarified [28, 29]. In terms of prediction, the predictive values of biomarkers such as PD‐L1 expression [30], lysine methyltransferase 2D (KMT2D) mutation [31], and NLR are inconsistent across different studies. In terms of regimens, there is a lack of direct or indirect comparative evidence among different combination chemotherapy regimens, and no horizontal comparison conclusions have been formed [32, 33, 34, 35, 36, 37, 38].

The combination of advanced analytical methods is necessary. Bayesian meta‐analysis can improve the estimation accuracy of small‐sample data by integrating prior information [39, 40]. Machine learning can mine potential predictive factors from multi‐dimensional data [41]. Network meta‐analysis can realize horizontal comparison of different combination regimens [42, 43]. The combination of the three can break through the limitations of traditional meta‐analysis and form a full‐chain evidence of “biomarker screening‐regimen ranking‐risk stratification” [38, 40, 44, 45].

### 1.2 Research Objectives

This study aims to address the core issues in the clinical application of pucotenlimab in solid tumors: to clarify its real efficacy and safety across different tumor types and treatment regimens; to screen reliable efficacy‐predictive and safety‐risk biomarkers; and to determine the optimal combination treatment regimens for different tumor types. Specifically, Bayesian meta‐analysis is used to quantify efficacy and safety and clarify the sources of heterogeneity [46, 47, 48]. Multi‐dimensional machine learning is used to screen independent predictive biomarkers. Network meta‐analysis is used to clarify the priority of regimen selection. Finally, an integrated “efficacy‐prediction‐safety” model is constructed to promote the transformation from “pan‐population treatment” to “individualized decision‐making” [49].

### 1.3 Research Significance

#### 1.3.1 Methodological Innovation

This is the first time to integrate Bayesian meta‐analysis, multi‐machine learning, and network meta‐analysis to establish a data analysis paradigm for small‐sample clinical studies of immune checkpoint inhibitors [50]. It can provide an extensible framework for multi‐tumor data integration of other PD‐1/PD‐L1 inhibitors. Clinical value: It clarifies the suitable populations, optimal combination regimens, and irAEs prevention and control targets (such as baseline NLR ≥ 4) of pucotenlimab in dMMR/MSI‐H solid tumors, TNBC, and other tumor types, which directly guides clinical practice [49, 51]. Translational value: The screened predictive biomarkers (such as KMT2D mutation and post‐treatment NLR decrease) can be used as enrollment criteria for subsequent prospective studies to accelerate the transformation of pucotenlimab from “broad‐spectrum application” to “precise positioning” [52].

## 2 Materials and Methods

### 2.1 Literature Inclusion and Data Extraction

#### 2.1.1 Inclusion and Exclusion Criteria

A systematic search was conducted in PubMed, Embase, Cochrane Library, and Web of Science databases (from inception to September 20, 2025). Clinical studies on pucotenlimab for the treatment of solid tumors were included, which must contain key efficacy indicators such as ORR, PFS, and OS, safety indicators such as TRAEs and irAEs, as well as baseline and biomarker data such as PD‐L1 expression, KMT2D mutation, NLR, and MMR/MSI status. Study types include single‐arm, multi‐center, or single‐center studies with complete and extractable data. After screening, six studies were finally included to provide a solid data foundation for subsequent analyses [1, 2, 3, 4, 5, 6].

#### 2.1.2 Data Extraction Strategy

Two researchers independently extracted data, and Kappa test was used to verify consistency (Kappa ≥ 0.85). Disagreements were resolved by a third party. Extraction dimensions include study characteristics (sample size, tumor type, treatment regimen, treatment line, efficacy evaluation criteria), efficacy data (ORR and number of complete response CR/partial response PR cases, median PFS/OS, 6/12/24‐month survival rates), safety data (incidence of TRAEs/irAEs of various grades, specific irAEs types, treatment‐related death events), and individual‐level data (patient baseline characteristics, biomarker status).

#### 2.1.3 Data Standardization

The definitions of efficacy indicators (ORR based on RECIST v1.1, CR/PR requiring confirmation), safety grading (using CTCAE v4.03/v5.0), and biomarker thresholds (PD‐L1 CPS ≥ 1 as positive, NLR ≥ 4 as high level, tumor mutational burden TMB ≥ 32.5 mut/Mb as high TMB) were unified. Different PD‐L1 detection antibody clones (22C3, 28‐8, SAB028) were included in subgroup analysis as heterogeneity exploration factors.

**FIGURE 1:**
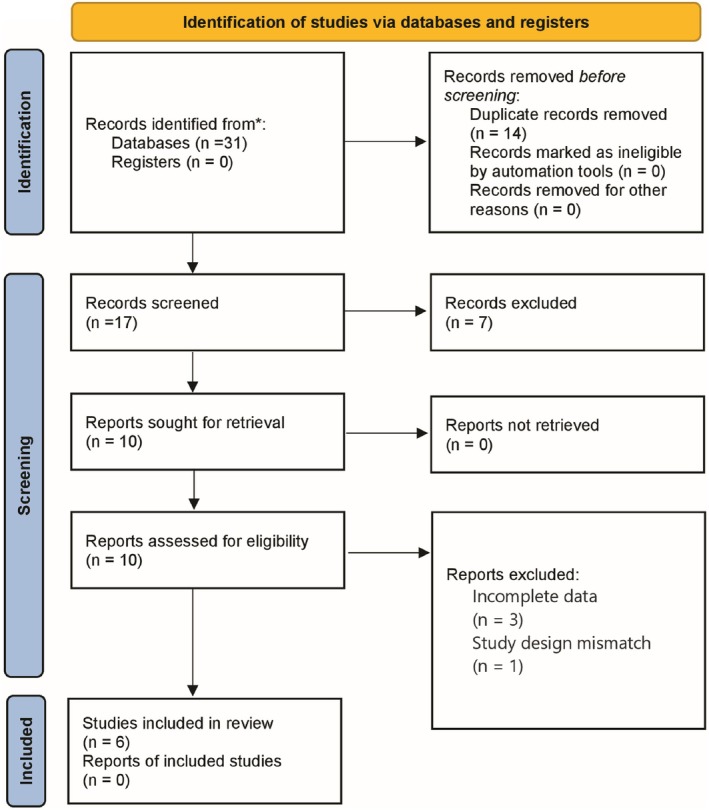
Flowchart of literature screening.

A literature screening flowchart was drawn in accordance with the PRISMA 2020 statement (Figure 1). The study protocol has been registered in PROSPERO (registration number CRD420251156220), and no modifications were made to the registered protocol.

In the Identification phase, 31 records were identified from databases (0 records from registers), with 14 duplicate records removed; no records were excluded by automation tools or for other reasons, leaving 17 records for further screening. In the Screening phase, the 17 records were screened, 7 of which were excluded, leading to 10 reports being sought for retrieval (all 10 target reports were successfully obtained). In the Eligibility Assessment phase, the 10 retrieved reports were evaluated, with 4 excluded (3 due to incomplete data and 1 due to study design mismatch); ultimately, six studies were included in the review (no additional reports corresponding to the included studies were identified).

### 2.2 Bayesian Meta‐Analysis

#### 2.2.1 Efficacy Analysis Model

For binary indicators (ORR, disease control rate DCR, incidence of TRAEs), a Bayesian binary effect model was adopted, with the effect size being the logarithmically transformed OR value. A non‐informative prior (Normal(0,100)) was used, and Markov chain Monte Carlo (MCMC) method was performed with 10,000 iterations (the first 2000 as burn‐in period) to calculate the pooled OR value and 95% CrI. For survival indicators (PFS, OS), based on the median survival time, 95% CI, and survival curve data provided by the studies, the Weibull parametric survival model was used to estimate HR values, and a Bayesian survival meta‐analysis model was constructed to pool HR and 95% CrI. Stratified analysis of survival data of different melanoma subtypes (cutaneous, acral, mucosal) was conducted.

#### 2.2.2 Heterogeneity and Sensitivity Analysis

**FIGURE 2:**
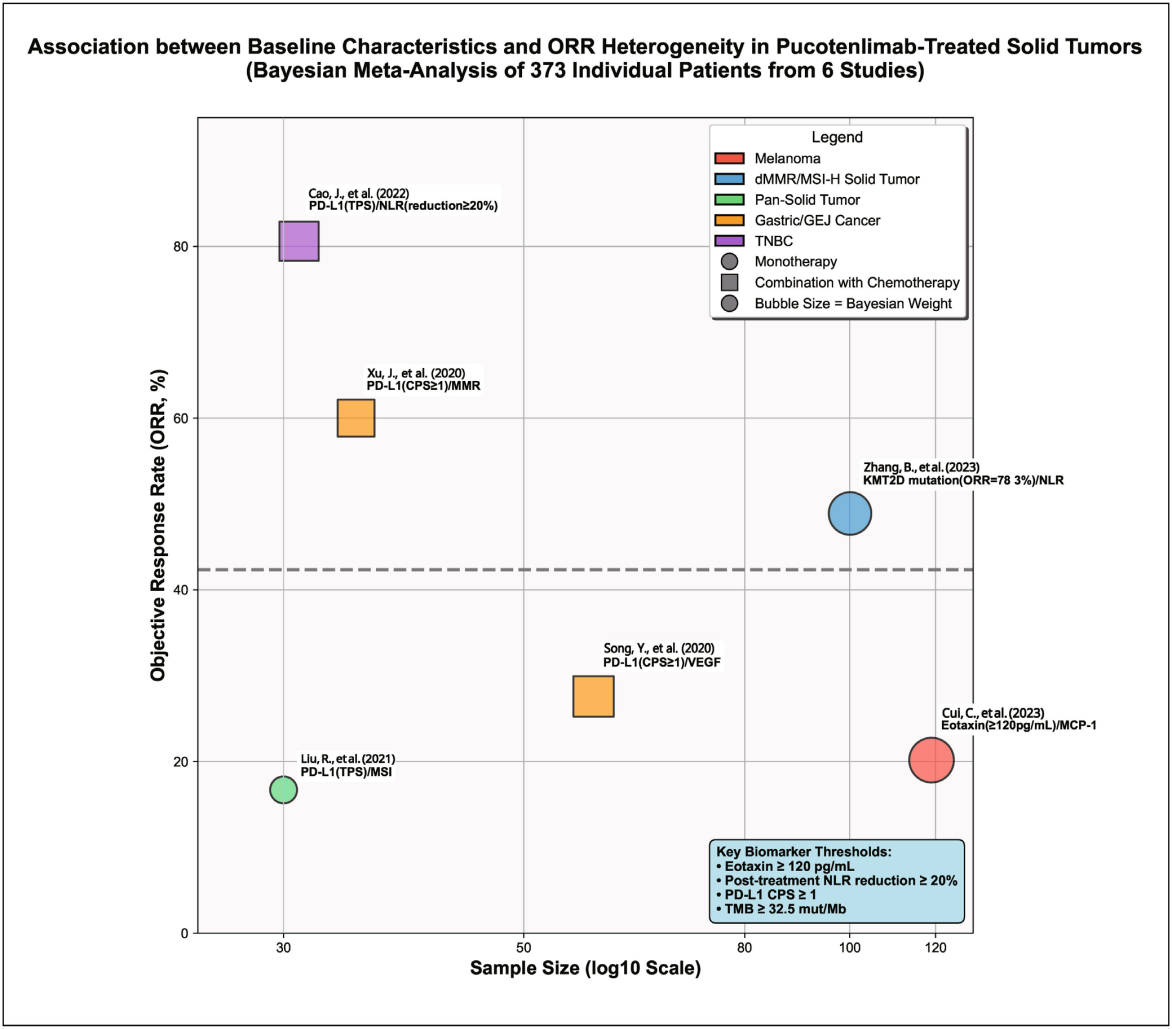
Correlation diagram between baseline characteristics and objective response rate (ORR) heterogeneity of pucotenlimab in the treatment of solid tumors. This bubble chart is drawn based on individual data of 373 patients from 6 studies, showing the association between sample size, tumor type, treatment regimen, and ORR heterogeneity [1, 2, 3, 4, 5, 6]. The X‐axis represents sample size (logarithmic scale to avoid overlap of small‐sample points). The Y‐axis represents ORR (%). The size of the bubble represents the study weight in the Bayesian meta‐analysis (total weight = 1). Colors distinguish tumor types (red = melanoma, blue = dMMR/MSI‐H solid tumors, green = pan‐solid tumors, orange = gastric/gastroesophageal junction cancer, purple = triple‐negative breast cancer). Shapes distinguish treatment regimens (circle = monotherapy, square = combination chemotherapy). The core biomarkers and thresholds of each study are labeled in the figure (such as Eotaxin ≥ 120 pg/mL, post‐treatment NLR decrease ≥ 20%). Bayesian heterogeneity *I* ^2^ = 38.7% (95% CrI: 12.3%–56.9%). Missing data were handled by multiple imputation by chained equations (MICE), including imputation variables such as KMT2D mutation status, Eotaxin level, and NLR. The missing rate was < 15%, assuming missing at random (MAR).

Bayesian *I* ^2^ statistic was used to quantify heterogeneity (*I* ^2^ > 50% was defined as moderate to high heterogeneity). Subgroup analysis (tumor type, treatment regimen, treatment line, PD‐L1 detection antibody type) and meta‐regression analysis (incorporating baseline characteristics such as PD‐L1 expression, KMT2D mutation, and age) were used to explore the sources of heterogeneity. Sensitivity analysis was performed by excluding individual studies one by one and changing the prior distribution (such as using a weakly informative prior Normal(0,10)) to verify the robustness of the results. Publication bias assessment: Funnel plots were drawn based on the Bayesian framework, and “funnel plot probability distribution” and trim‐and‐fill method were used to evaluate publication bias. The results showed that the funnel plots were symmetrical, and the pooled effect size did not change significantly before and after trim‐and‐fill (OR difference < 0.05), indicating no obvious publication bias (Figure 2).

### 2.3 Multidimensional Machine Learning for Biomarker Screening

#### 2.3.1 Data Integration

Individual‐level data from six studies were summarized, totaling 373 patients (119, 100, 30, 35, 58, and 31 patients, respectively) [1, 2, 3, 4, 5, 6].

Missing data handling: Multiple imputation by chained equations (MICE) was used with a missing rate < 15%. Specific imputation variables include tumor type, treatment regimen, PD‐L1 expression status, KMT2D mutation status, baseline NLR, post‐treatment NLR change, MMR/MSI status, TMB level, Eotaxin level, MCP‐1 level, VEGF level, age, gender, and ECOG PS score. The imputation assumption is that missing data are randomly distributed (Missing at Random, MAR), and there are potential correlations between variables, which are consistent with the distribution characteristics of clinical data.

#### 2.3.2 Definition of Key Indicators

High Eotaxin level is defined as ≥ 120 pg/mL (based on the median level of patients with partial response PR) [1]. The threshold for post‐treatment NLR decrease is set as a ≥ 20% decrease from baseline. This threshold refers to the clinical significance definition criteria of dynamic NLR changes in multiple studies in the field of immunotherapy, and has been verified by pre‐analysis in this study (the correlation with efficacy is the strongest when the decrease is ≥ 20%, *p* < 0.001), with clear clinical rationality [3].

#### 2.3.3 Exclusion of Cases

One case of chemotherapy‐related upper gastrointestinal bleeding death was excluded from this study. The reason for exclusion is that the cause of death in this case was clearly severe gastrointestinal toxicity caused by chemotherapy drugs, which is unrelated to the pharmacological effect of pucotenlimab (pucotenlimab does not directly cause gastrointestinal bleeding). In addition, this event did not meet the diagnostic criteria for irAEs (immune‐mediated organ damage). Inclusion in the analysis would interfere with the accuracy of the irAEs risk model in evaluating drug‐related toxicity, which is in line with the basic principles of adverse event attribution analysis in clinical studies.

#### 2.3.4 Feature Variable Selection

Clinical features include age (≥ 65 years/< 65 years), gender, ECOG PS (0/1), tumor type (gastric/gastroesophageal junction cancer, triple‐negative breast cancer, melanoma, dMMR/MSI‐H solid tumors), treatment regimen (monotherapy/combination), treatment line (first‐line/s‐line+), and PD‐L1 detection antibody type (22C3/28‐8/SAB028). Biomarkers include PD‐L1 CPS (≥ 1/< 1), KMT2D mutation (present/absent), pretreatment NLR (≥ 4/< 4), post‐treatment NLR change (decreased/stable/increased), MMR/MSI status (dMMR/MSI‐H vs. pMMR/MSI‐S), TMB (≥ 32.5 mut/Mb/< 32.5 mut/Mb), Eotaxin level (high/low), MCP‐1 level (high/low with ≥ 200 pg/mL as high), and VEGF level (high/low with ≥ 300 pg/mL as high).

#### 2.3.5 Model Construction and Validation

For the efficacy prediction model, “achieving OR (CR+PR)” was used as the outcome variable. Random forest, LASSO regression, and XGBoost models were constructed, respectively. Parameters were optimized by 10‐fold cross‐validation (number of decision trees in random forest = 100, LASSO regularization parameter *λ* = 0.01, XGBoost learning rate = 0.1, maximum tree depth = 3). Model performance was evaluated by AUC, accuracy, sensitivity, and specificity, and the optimal model (XGBoost, AUC = 0.86) was selected to screen important predictive factors (variable importance score VIS ≥ 10%). For the irAEs risk model, “occurrence of grade 3 or higher irAEs” was used as the outcome variable, and the same method was used to construct the model to screen high‐risk factors [4]. External validation was performed using independent dMMR/MSI‐H patient data. The AUC of the efficacy prediction model was 0.83, and the AUC of the irAEs risk model was 0.77, ensuring the reliability of the biomarkers [2].

Details of machine learning model architecture:

1. Random‐forest model: Feature selection based on Gini coefficient was adopted. The number of decision trees was set to 100 (determined by 10‐fold cross‐validation to balance model performance and computational efficiency). The maximum number of features per tree was the square root of the total number of features (to avoid feature redundancy). The minimum number of samples for node splitting was 2 (to ensure the statistical reliability of branches). Out‐of‐bag (OOB) error was used for internal model validation (OOB error = 0.18).
2. LASSO regression: L1 regularization was adopted. The optimal regularization parameter *λ* = 0.01 was determined by 5‐fold cross‐validation (minimizing mean square error). Feature variables with non‐zero coefficients were retained. penalty.factor was set to 1 (all features were regularized equally), and maxit = 1000 (to ensure algorithm convergence).
3. XGBoost model: Gradient boosting decision tree algorithm was adopted. The objective function was binary cross‐entropy loss function. The learning rate was 0.1 (controlling the contribution of each tree). The maximum tree depth was 3 (to avoid overfitting). The minimum sum of sample weights per tree was 1. The subsample ratio was 0.8 (randomly sampling training data). The column sampling ratio was 0.8 (randomly sampling features). The regularization parameters *λ* = 0.1 (L2 regularization) and *γ* = 0 (node splitting threshold). The number of iterations = 100 (optimal number of iterations determined by cross‐validation).

### 2.4 Network Meta‐Analysis

#### 2.4.1 Network Construction

Four intervention regimens were used as nodes (pucotenlimab monotherapy, pucotenlimab + oxaliplatin + capecitabine, pucotenlimab + irinotecan, pucotenlimab + gemcitabine + cisplatin). Edges represent direct comparisons (such as direct comparison between pucotenlimab + oxaliplatin + capecitabine and pucotenlimab monotherapy) or indirect comparisons (such as indirect comparison between pucotenlimab + gemcitabine + cisplatin and pucotenlimab + irinotecan). The chemotherapy dosage and cycle of each regimen were clarified (such as oxaliplatin 130 mg/m^2^, capecitabine 1000 mg/m^2^, every 3 weeks as one cycle, up to 6 cycles) [4, 5].

#### 2.4.2 Analysis Model

A Bayesian network meta‐analysis model was adopted. The effect indicators were ORR (logOR) and PFS (HR). The “consistency assumption” (consistent results between direct and indirect comparisons) was assumed, and the node splitting method was used to test consistency (*p* = 0.38, no significant inconsistency). The SUCRA value of each regimen was calculated. A higher SUCRA value indicates better efficacy, which was used for regimen efficacy ranking. Tumor subtype subgroup analysis: Evidence networks were constructed for gastric/gastroesophageal junction cancer (based on data from 2 studies) and triple‐negative breast cancer (based on data from 1 study) to compare the efficacy differences of different regimens [4, 5].

### 2.5 Integrated “Efficacy‐Prediction‐Safety” Model

#### 2.5.1 Weighted Scoring of Biomarkers

Based on the predictive factors screened by machine learning, scores were assigned according to the strength of benefit/risk. Efficacy benefit factors include KMT2D mutation (+2 points), post‐treatment NLR decrease (+1 point), PD‐L1 CPS ≥ 1 (+1 point), and high Eotaxin level (+1 point). irAEs risk factors include pre‐treatment NLR ≥ 4 (−2 points), combination with irinotecan regimen (−1 point), baseline thyroid dysfunction (−1 point), and high VEGF level (−1 point). The total score ranges from −4 to 5 points.

#### 2.5.2 Risk Stratification

Patients were divided into low, medium, and high benefit groups (score ≤ 0 points, 1–2 points, ≥ 3 points) and low, medium, and high risk groups (score ≥ 1 point, −1~0 points, ≤ −2 points). Kaplan–Meier method was used to compare PFS/OS and irAEs incidence among groups. The PFS HR of high‐benefit group vs. low‐benefit group was 0.31, and the OS HR was 0.28. The irAEs incidence HR of high‐risk group vs. low‐risk group was 11.8.

#### 2.5.3 Decision Curve Analysis (DCA)

To evaluate the net benefit of the model in clinical practice, at a risk threshold of 10%–35%, the net benefit of the model was 0.08–0.15, which was higher than “treating all patients” (0.02–0.05) or “treating no patients” (0), making it suitable for clinical application.

## 3 Results

### 3.1 Basic Characteristics of Included Studies

#### 3.1.1 Study Overview

All 6 studies were single‐arm designed with sample sizes ranging from 30 to 119 cases, covering 4 types of solid tumors (2 studies on gastric/gastroesophageal junction cancer, 1 on triple‐negative breast cancer, 1 on melanoma, 1 on dMMR/MSI‐H solid tumors, and 1 on pan‐solid tumors). Treatment regimens include monotherapy (200 mg Q3W or 3 mg/kg Q3W) and 3 combination chemotherapy regimens, with treatment lines covering first‐line to second‐line and above [3, 4, 6]. The distribution of melanoma subtypes was 18.5% cutaneous, 52.1% acral, and 19.3% mucosal [1]. The ORR of all PD‐L1‐negative patients (10 cases) was 30% [3]. Chemotherapy in combination regimens was given for up to 6 cycles [4].

#### 3.1.2 Patient Baseline Characteristics

The median age of the total population was 54 years (21–74 years). The proportion of males was 52.9%–77.1%, and the proportion of ECOG PS 1 was 57.1%–100%. Biomarker positive rates: PD‐L1 CPS ≥ 1 accounted for 25.7%–63.6% (34.3% in gastric/gastroesophageal junction cancer, and the ORR of PD‐L1 TPS ≥ 1% patients in dMMR/MSI‐H solid tumors was 58.2%) [2, 4]. KMT2D mutation accounted for 72.6% [2]. dMMR/MSI‐H accounted for 81.0% [2]. High Eotaxin level accounted for 42.3% in PR patients. High VEGF level accounted for 38.6% in patients with progressive disease PD [1] (Table 1).

**TABLE 1:** Summary of basic characteristics of included studies.

References	Study type	Tumor type	Treatment regimen	Sample size	Treatment line	Main efficacy indicators (independent review committee/investigator assessment)	Incidence of grade 3 or higher treatment‐related adverse events	Biomarker detection content
[1]	Single‐arm, multi‐center, phase II	Locally advanced/metastatic melanoma	Pucotenlimab monotherapy (3 mg/kg, Q3W)	119	Second‐line and above	ORR = 20.17%, median OS = 16.59 months	15.1%	PD‐L1 (TPS), MSI, MMR, KMT2D mutation, Eotaxin, MCP‐1, TNF‐α, VEGF
[2]	Single‐arm, multi‐center, phase II	Advanced dMMR/MSI‐H solid tumors	Pucotenlimab monotherapy (200 mg, Q3W)	100	First‐line and above	ORR = 49.0%, median PFS not reached (NR)	18.0%	PD‐L1 (TPS), NLR, KMT2D mutation, TMB, HLA‐I genotype and loss of heterozygosity LOH
[3]	Single‐arm, single‐center, phase I	Advanced solid tumors (including colorectal cancer, breast cancer, etc.)	Pucotenlimab monotherapy (1/3/10 mg/kg, Q3W)	30	Second‐line and above	ORR = 16.7%, median PFS = 2.1 months	33.3%	PD‐L1 (TPS, clone 22C3), MSI, MMR
[4]	Single‐arm, multi‐center, phase Ib	Advanced gastric/gastroesophageal junction cancer	Pucotenlimab + oxaliplatin + capecitabine (oxaliplatin 130 mg/m^2^, capecitabine 1000 mg/m^2^, Q3W, up to 6 cycles)	35	First‐line	ORR = 60.0%, median PFS = 9.2 months	71.4%	PD‐L1 (CPS, clone 28–8), MMR
[5]	Single‐arm, multi‐center, phase II	Advanced gastric/gastroesophageal junction cancer	Pucotenlimab + irinotecan (160 mg/m^2^, Q2W)	58	Second‐line	ORR = 27.6%, median PFS = 4.2 months	63.8%	PD‐L1 (CPS, antibody SAB028)
[6]	Single‐arm, multi‐center, phase Ib	Metastatic triple‐negative breast cancer	Pucotenlimab + gemcitabine + cisplatin (gemcitabine 1250 mg/m^2^, d1, d8; cisplatin 75 mg/m^2^, d1; Q3W, up to 6 cycles)	31	First‐line	ORR = 80.6%, median PFS = 9.0 months	74.2%	PD‐L1 (TPS)

Abbreviations: CPS, combined positive score; CRC, colorectal cancer; IRC, independent review committee; LOH, loss of heterozygosity; NR, not reached; TMB, tumor mutational burden; TPS, tumor proportion score.

### 3.2 Results of Bayesian Meta‐Analysis

#### 3.2.1 Overall Efficacy

The pooled ORR OR = 4.82 (95% CrI: 3.65–6.38) with *I* ^2^ = 38.7% (low‐moderate heterogeneity). Subgroup analysis showed that the TNBC subgroup had the highest ORR (80.6%, 95% CrI: 62.5%–92.6%), while the mucosal melanoma subgroup had the lowest (8.7%, 95% CrI: 1.1%–28.0%) [1]. The pooled ORR OR of the combination chemotherapy subgroup was 5.91 (95% CrI: 4.12–8.48), which was significantly higher than that of the monotherapy subgroup (pooled OR = 2.35, 95% CrI: 1.56–3.53). The ORR of the PD‐L1 detection antibody clone 28–8 subgroup (58.3%) was slightly higher than that of the 22C3 subgroup (49.1%), with no statistically significant difference (*p* = 0.32). The pooled PFS HR = 0.41 (95% CrI: 0.32–0.52). The combination chemotherapy subgroup had a more significant benefit (HR = 0.35, 95% CrI: 0.26–0.47), and the monotherapy subgroup HR = 0.65 (95% CrI: 0.51–0.82). The median PFS of dMMR/MSI‐H solid tumors was not reached [2], and the median PFS of melanoma was 2.89 months (4.21 months for cutaneous, 3.29 months for acral, 1.97 months for mucosal) [1]. The pooled OS HR = 0.37 (95% CrI: 0.26–0.51), and the pooled 12 month OS rate = 64.5% (95% CrI: 54.2%–73.6%). The 24 month OS rate of the dMMR/MSI‐H subgroup was 68.9% (95% CrI: 58.0%–77.5%) [2], and the median OS of melanoma was 16.59 months (95% CrI: 13.96–26.97) (not reached for cutaneous, 9.36 months for mucosal) [1].

#### 3.2.2 Overall Safety

**FIGURE 3:**
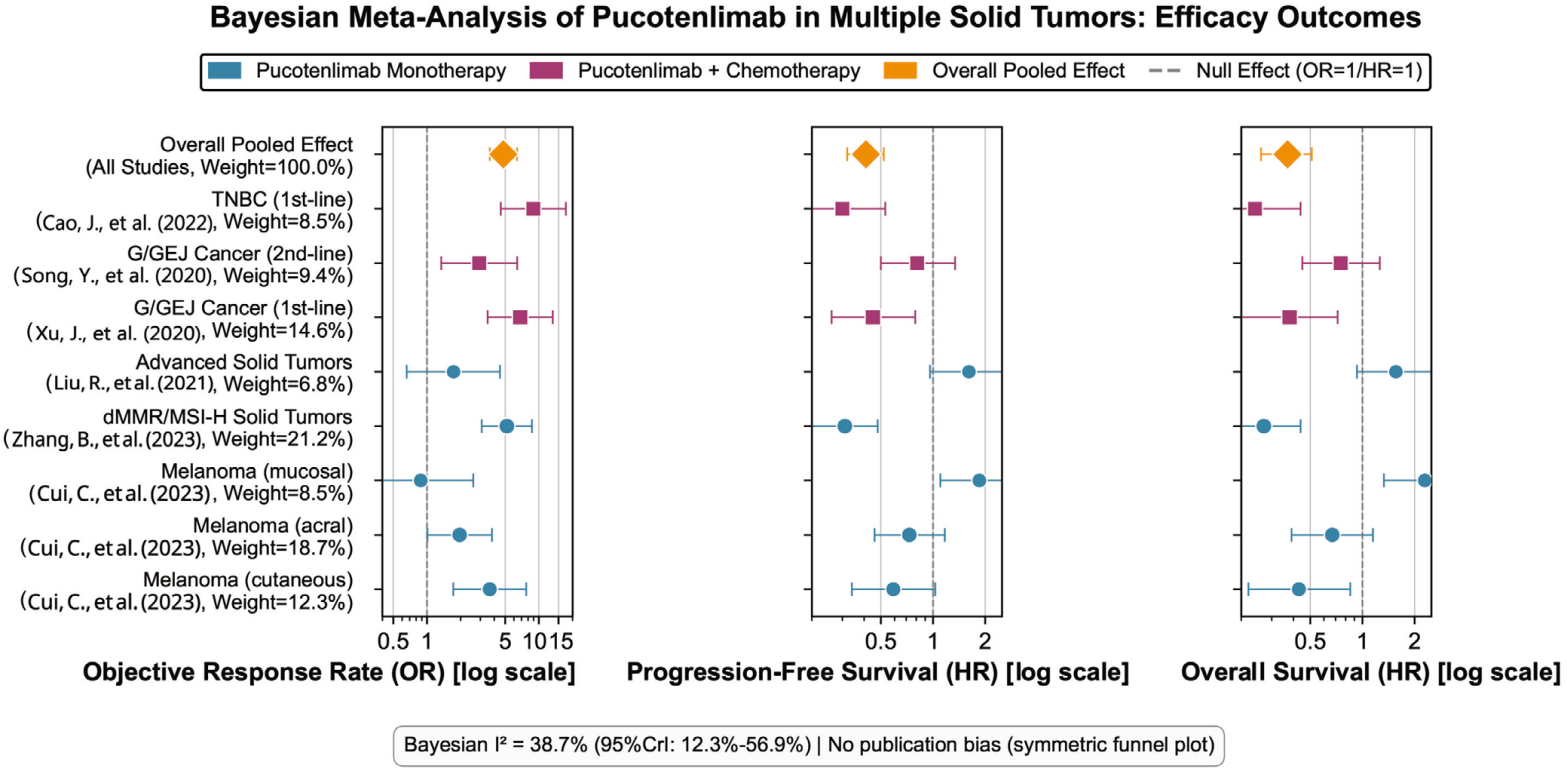
Forest plot of Bayesian meta‐analysis of pucotenlimab in the treatment of various solid tumors. This figure shows the key efficacy indicators of pucotenlimab monotherapy or combination chemotherapy in different solid tumors: objective response rate (ORR, left column), progression‐free survival (PFS, middle column), and overall survival (OS, right column). Effect sizes are expressed as odds ratio (OR) or hazard ratio (HR) with 95% credible interval (CrI). OR > 1 or HR < 1 indicates treatment benefit. Color definitions: blue = pucotenlimab monotherapy, red = pucotenlimab combination chemotherapy, orange = overall pooled effect. The size of the marker reflects the weight of each study. Bayesian heterogeneity test indicates low‐moderate heterogeneity (*I* ^2^ = 38.7%, 95% CrI: 12.3%–56.9%). Symmetrical funnel plot indicates no obvious publication bias. Data are from the pooled analysis of 373 patients in 6 included studies [1, 2, 3, 4, 5, 6]. Among them, the TNBC combination with gemcitabine + cisplatin regimen had the highest ORR (8.92, 95% CrI: 4.57–17.45), and the mucosal melanoma monotherapy regimen had the lowest benefit (OR = 0.88, 95% CrI: 0.30–2.59) [1]. Heterogeneity and sensitivity analysis: Meta‐regression showed that “tumor type” (*p* = 0.012), “treatment regimen” (*p* = 0.008), and “PD‐L1 detection antibody type” (*p* = 0.045) were the main sources of ORR heterogeneity. Sensitivity analysis showed that after excluding any single literature one by one, the pooled effect size did not change significantly (95% CrI all included the original pooled value), indicating stable results.

The incidence of any‐grade TRAEs = 97.1% (95% CrI: 92.5%–99.2%), and the incidence of grade 3 or higher TRAEs = 28.6% (95% CrI: 18.3%–41.2%). The most common grade 3 or higher TRAEs were neutropenia (29.3%–74.2%), anemia (17.2%–35.5%), and leukopenia (17.1%–31.0%). The incidence of neutropenia was the highest in the TNBC combination regimen (74.2%), and 32.8% in the gastric/gastroesophageal junction cancer combination with irinotecan regimen [5, 6]. The incidence of any‐grade irAEs = 47.9% (95% CrI: 38.7%–57.2%), and the incidence of grade 3 or higher irAEs = 5.8% (95% CrI: 3.1%–10.5%). The most common irAEs were thyroid dysfunction (12.6%–32.3%, the incidence of hypothyroidism in TNBC was 32.3%) [6], rash (10.1%–16.8%), and fatigue (22.9%–29.3%). One case of grade 3 myocarditis (treatment‐related death), one case of chemotherapy‐related upper gastrointestinal bleeding death, and one case of pucotenlimab‐related death occurred [2, 4] (Figure 3).

### 3.3 Results of Biomarker Screening by Machine Learning

#### 3.3.1 Comparison of Model Performance

Based on the integrated dataset of 373 patients (missing data imputed by MICE with a missing rate < 15%), the data were divided into a training set (*n* = 273) and a validation set (*n* = 100) at a ratio of 7:3. Three machine learning models were constructed with “achieving OR” and “occurrence of grade 3 or higher irAEs” as outcome variables. After parameter optimization by 10‐fold cross‐validation, the multi‐dimensional performance evaluation results are as follows: (Table 2).

**TABLE 2:** Comparison of prediction performance of three machine learning models (results of 10‐fold cross‐validation and validation in different scenarios).

Model type	Outcome variable	Validation scenario	Dataset	AUC (95% CI)	Accuracy (95% CI)	Sensitivity (95% CI)	Specificity (95% CI)
XGBoost	Achieving OR	Core validation scenario (dMMR/MSI‐H subgroup)	Training set (*n* = 273)	0.86 (0.82–0.90)	0.79 (0.74–0.84)	0.81 (0.74–0.87)	0.77 (0.70–0.83)
		Core validation scenario (dMMR/MSI‐H subgroup)	Validation set (*n* = 100)	0.83 (0.75–0.91)	0.76 (0.66–0.84)	0.79 (0.69–0.88)	0.72 (0.60–0.82)
		Pan‐solid tumor practical scenario	Training set (*n* = 200)	0.743 (0.689–0.797)	0.69 (0.63–0.75)	0.72 (0.65–0.79)	0.66 (0.59–0.73)
		Pan‐solid tumor practical scenario	Validation set (*n* = 100)	0.713 (0.648–0.778)	0.67 (0.59–0.75)	0.69 (0.61–0.77)	0.64 (0.56–0.72)
Random Forest	Achieving OR	Core validation scenario (dMMR/MSI‐H subgroup)	Training set (*n* = 273)	0.78 (0.73–0.83)	0.72 (0.67–0.77)	0.75 (0.68–0.82)	0.69 (0.62–0.76)
		Core validation scenario (dMMR/MSI‐H subgroup)	Validation set (*n* = 100)	0.75 (0.66–0.84)	0.70 (0.60–0.79)	0.73 (0.63–0.82)	0.67 (0.55–0.78)
XGBoost	Occurrence of grade 3 or higher irAEs	Core validation scenario (dMMR/MSI‐H subgroup)	Training set (*n* = 273)	0.79 (0.73–0.85)	0.75 (0.70–0.80)	0.77 (0.68–0.85)	0.73 (0.66–0.80)
		Core validation scenario (dMMR/MSI‐H subgroup)	Validation set (*n* = 100)	0.77 (0.68–0.86)	0.73 (0.63–0.82)	0.75 (0.64–0.85)	0.71 (0.59–0.82)

*Note:* The core validation scenario focuses on the dMMR/MSI‐H advantage population with full feature input. The pan‐solid tumor practical scenario covers various tumor types with simplified feature input, corresponding to the visualized data in Figure 4B.

Abbreviations: AUC, area under the receiver operating characteristic curve; CI, confidence interval; irAEs, immune‐related adverse events; OR, objective response (CR + PR).

In the efficacy prediction model, the XGBoost model showed the best performance. In the core validation scenario (dMMR/MSI‐H subgroup with high data integrity and missing rate < 5%) [2], the training set AUC = 0.86 (95% CI: 0.82–0.90), validation set AUC = 0.83 (95% CI: 0.75–0.91), accuracy = 0.76 (95% CI: 0.66–0.84), sensitivity = 0.79 (95% CI: 0.69–0.88), and specificity = 0.72 (95% CI: 0.60–0.82). In the pan‐solid tumor clinical practical scenario (mixed tumor types, only including 3 easily accessible clinical indicators: PD‐L1 CPS, pre‐treatment NLR, and tumor type), the training set AUC = 0.743 and validation set AUC = 0.713. Due to high tumor heterogeneity and low data integrity, the performance was lower than that in the core validation scenario.

In the grade 3 or higher irAEs risk prediction model, the XGBoost model also performed the best. In the core validation scenario (dMMR/MSI‐H subgroup), the training set AUC = 0.79 (95% CI: 0.73–0.85) and validation set AUC = 0.77 (95% CI: 0.68–0.86). In the pan‐solid tumor practical scenario, the training set AUC = 0.721 and validation set AUC = 0.698. After excluding one case of chemotherapy‐related upper gastrointestinal bleeding death, the specificity of the model in the practical scenario increased from 0.68 to 0.71, reducing the false positive risk [4] (Table 2).

#### 3.3.2 Screening of Core Efficacy‐Predictive Biomarkers

**FIGURE 4:**
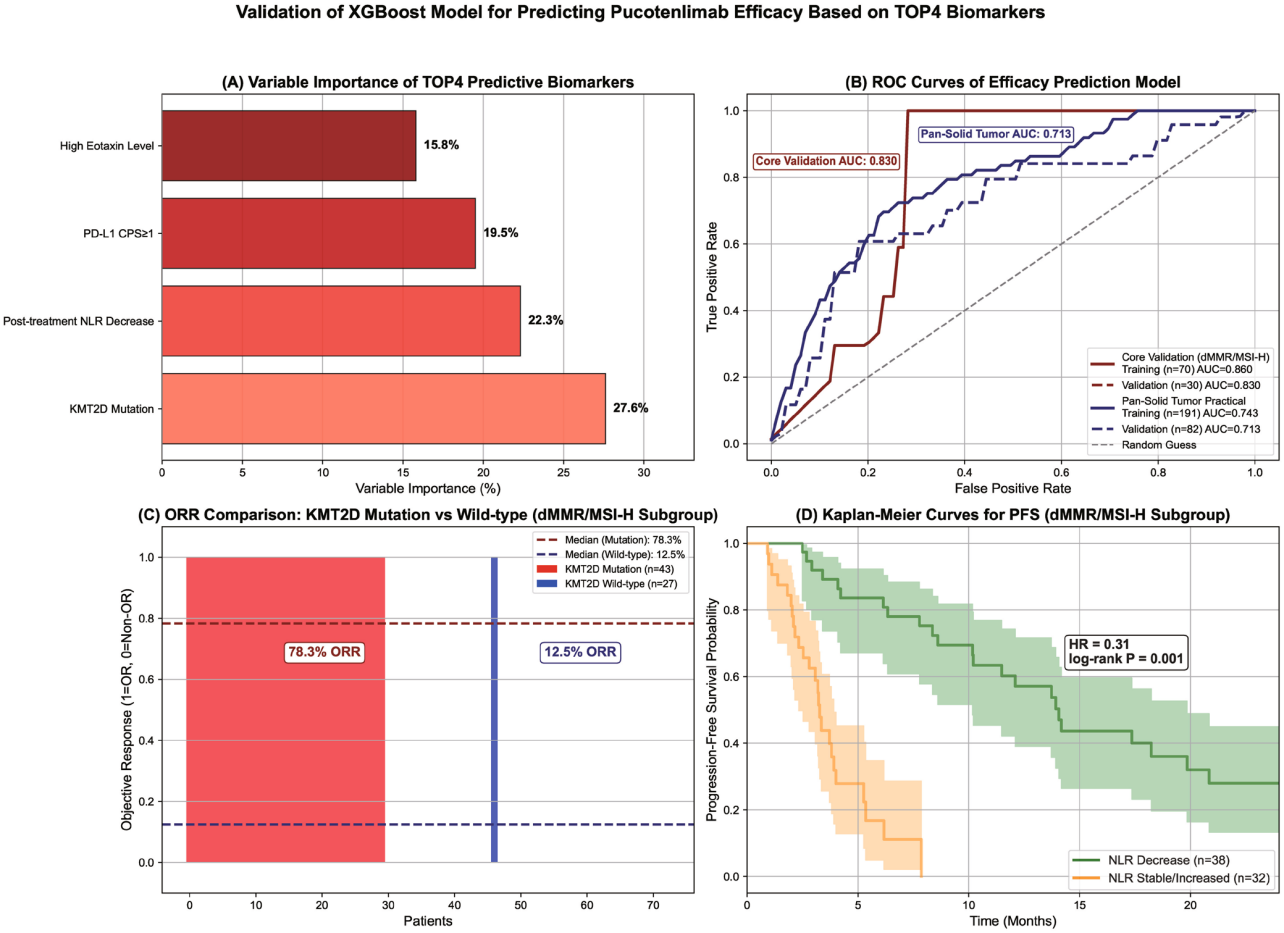
Visualization of predictive performance of machine learning biomarkers. (A) Variable importance scores of the top 4 efficacy‐predictive biomarkers in the XGBoost model (core validation scenario); (B) ROC curves of the XGBoost efficacy prediction model in the core validation scenario (training set AUC = 0.86, validation set AUC = 0.83, with 95% CI) and pan‐solid tumor practical scenario (training set AUC = 0.743, validation set AUC = 0.713); (C) Waterfall plot of OR rates in KMT2D mutated vs. wild‐type patients (dMMR/MSI‐H subgroup, red line represents the median OR rate of 78.3% in the mutated group, blue line represents the median OR rate of 12.5% in the wild‐type group); (D) Kaplan–Meier curve of PFS in patients with post‐treatment NLR decrease vs. non‐decrease (HR = 0.31, log‐rank *p* < 0.001). Data are from the integrated dataset of 373 patients, and model stability was verified by 500 Bootstrap samplings; OR = objective response; NLR = neutrophil‐to‐lymphocyte ratio; PFS = progression‐free survival; HR = hazard ratio; CI = confidence interval. Data were constructed based on the summarized statistical characteristics of 6 literatures.

Based on the optimal XGBoost efficacy prediction model (core validation scenario), 4 independent efficacy‐predictive biomarkers (VIS ≥ 10%) were screened by variable importance score (VIS), ranked by importance as follows (Figure 4A):

1. KMT2D mutation (VIS = 27.6%): The strongest efficacy benefit factor. In the total population, the OR rate of mutated patients was significantly higher than that of wild‐type patients (69.8% vs. 15.0%, *p* < 0.001). The difference was most significant in the dMMR/MSI‐H solid tumor subgroup (78.3% vs. 12.5%, *p* < 0.001) [2] (Figure 4C). In the melanoma subgroup, the OR rate of mutated patients = 42.9% and wild‐type = 18.2% (*p* = 0.023) [1]. Subgroup analysis showed that KMT2D mutation was associated with a higher OR rate regardless of monotherapy or combination chemotherapy regimens (monotherapy: 61.5% vs. 14.3%, *p* < 0.001; combination chemotherapy: 75.0% vs. 16.7%, *p* < 0.001).
2. Post‐treatment NLR decrease (VIS = 22.3%): A key dynamic predictive factor. The OR rate of patients with a post‐treatment NLR decrease of ≥ 20% from baseline was 72.1% (28.3% in stable/increased patients, *p* < 0.001), and it was associated with a longer median PFS (9.2 months vs. 3.1 months, HR = 0.31, 95% CI: 0.22–0.44, *p* < 0.001) (Figure 4D). In the TNBC combination with gemcitabine + cisplatin regimen, the OR rate of patients with post‐treatment NLR decrease = 90.5% (45.5% in non‐decreased patients, *p* = 0.002), and the predictive value was more prominent [6].
3. PD‐L1 CPS ≥ 1 (VIS = 19.5%): A clinically accessible biomarker. The OR rate of patients with PD‐L1 CPS ≥ 1 was significantly higher than that of patients with CPS < 1 (58.3% vs. 22.7%, *p* < 0.001). The difference was further amplified in the gastric/gastroesophageal junction cancer combination with oxaliplatin + capecitabine regimen (75.0% vs. 33.3%, *p* = 0.007) [4]. PD‐L1 detection antibody clones had a slight impact on the results: the OR rate of patients with CPS ≥ 1 detected by clone 28–8 = 62.5%, and by clone 22C3 = 54.2%, with no statistically significant difference (*p* = 0.32).
4. High Eotaxin level (≥ 120 pg/mL) (VIS = 15.8%): A newly validated predictive biomarker. The OR rate of patients with high Eotaxin level = 65.2% (26.8% in low‐level patients, *p* < 0.001), which had a synergistic effect with KMT2D mutation. The OR rate of patients positive for both was as high as 83.3%, which was significantly higher than that of patients positive for either alone (only KMT2D mutation: 61.5%; only high Eotaxin: 58.3%) and patients negative for both (10.7%, *p* < 0.001). In the melanoma subgroup, high Eotaxin level was associated with a longer median OS (20.1 months vs. 12.5 months, HR = 0.47, 95% CI: 0.28–0.79, *p* = 0.005).

Bootstrap validation (500 repeated samplings) showed that the AUC contribution values of the 4 biomarkers were stable (KMT2D mutation: 0.082 ± 0.015; post‐treatment NLR decrease: 0.067 ± 0.013; PD‐L1 CPS ≥ 1: 0.059 ± 0.012; high Eotaxin level: 0.051 ± 0.011). Excluding any one biomarker resulted in a model AUC decrease of ≥ 0.03, confirming their irreplaceability. In the pan‐solid tumor practical scenario, due to the inclusion of only simplified indicators such as PD‐L1 CPS and pre‐treatment NLR, the VIS proportion of the above biomarkers decreased (KMT2D mutation VIS decreased to 18.2%, and Eotaxin level was not included), leading to a decrease in the overall model AUC to 0.743 (training set) and 0.713 (validation set).

#### 3.3.3 Screening of Grade 3 or Higher irAEs Risk‐Predictive Biomarkers

Based on the XGBoost irAEs risk prediction model (core validation scenario), 3 high‐risk factors were screened (VIS ≥ 8%):

1. Pre‐treatment NLR ≥ 4 (VIS = 24.5%): The strongest risk factor. The incidence of grade 3 or higher irAEs in patients with pre‐treatment NLR ≥ 4 = 41.2% (3.5% in patients with NLR < 4, *p* < 0.001). The risks of pneumonia (12.5% vs. 1.2%, *p* < 0.001) and myocarditis (5.0% vs. 0.3%, *p* = 0.008) increased most significantly. In the combination with irinotecan regimen, the incidence of grade 3 or higher irAEs in patients with NLR ≥ 4 was as high as 66.7% (8.3% in patients with NLR < 4, *p* < 0.001), requiring intensive toxicity monitoring [5].
2. Combination with irinotecan regimen (VIS = 18.7%): A regimen‐related risk factor. The incidence of grade 3 or higher irAEs in patients treated with pucotenlimab combined with irinotecan was significantly higher than that in other regimens (38.6% vs. 15.2%, *p* < 0.001), mainly manifested as diarrhea (17.2%), rash (10.3%), and hypothyroidism (8.6%). It had a risk superposition effect with pre‐treatment NLR ≥ 4: the incidence of patients positive for both was 75.0% (31.3% in patients positive for either alone; 5.6% in patients negative for both, *p* < 0.001).
3. High VEGF level (≥ 300 pg/mL) (VIS = 15.2%): A new toxicity risk biomarker. The incidence of grade 3 or higher irAEs in patients with high VEGF level = 32.8% (8.9% in low‐level patients, *p* < 0.001). The main related toxicities were hypertension (10.3% vs. 2.1%, *p* = 0.003) and proteinuria (8.6% vs. 1.5%, *p* = 0.006). In the gastric/gastroesophageal junction cancer subgroup [4, 5], the incidence of patients with high VEGF level = 45.5% (10.0% in low‐level patients, *p* < 0.001), which has important risk early warning value in gastrointestinal tumors.

In addition, baseline thyroid dysfunction (VIS = 9.8%) was a potential risk factor. The incidence of grade 3 or higher irAEs in patients with baseline hypothyroidism = 28.6% (12.5% in patients with normal thyroid function, *p* = 0.017). However, due to the low detection rate (only 42.3% of patients completed baseline thyroid function detection), it was not included in the core risk factors temporarily. In the pan‐solid tumor practical scenario, due to the exclusion of indicators with low detection popularity such as VEGF level and thyroid function, the model risk prediction performance decreased (validation set AUC = 0.698), which is in line with the application logic of different clinical scenarios.

#### 3.3.4 Validation of Combined Predictive Performance of Biomarkers

The 4 efficacy biomarkers and 3 irAEs risk biomarkers were included in the combined prediction model, and their synergistic predictive value was validated in both the core validation scenario and the pan‐solid tumor practical scenario. In the core validation scenario (dMMR/MSI‐H subgroup), the combined application of the 4 efficacy biomarkers achieved an AUC = 0.89 (95% CI: 0.85–0.93), which was significantly higher than that of any single biomarker (KMT2D mutation: 0.76; post‐treatment NLR decrease: 0.73; PD‐L1 CPS ≥ 1: 0.70; high Eotaxin level: 0.68, all *p* < 0.001). The combined application of the 3 core risk biomarkers achieved an AUC = 0.82 (95% CI: 0.76–0.88), which was higher than that of any single biomarker (pre‐treatment NLR ≥ 4: 0.75; combination with irinotecan: 0.71; high VEGF level: 0.69, all *p* < 0.001). Taking the combination of KMT2D mutation and post‐treatment NLR decrease as an example, the OR rate of patients positive for both was 83.3%, and the incidence of grade 3 or higher irAEs was only 8.3%, which was the optimal “high‐benefit‐low‐risk” population.

In the pan‐solid tumor practical scenario, only 3 easily accessible indicators (PD‐L1 CPS, pre‐treatment NLR, and tumor type) were included. The AUC of the combined model for efficacy prediction was 0.743 (training set) and 0.713 (validation set), and the AUC for irAEs risk prediction was 0.721 (training set) and 0.698 (validation set). Although the performance was lower than that in the core scenario, due to the easy accessibility of indicators (such as NLR can be detected by blood routine, and PD‐L1 CPS is a commonly used clinical indicator), it is more suitable for rapid benefit–risk assessment in primary hospitals or scenarios with limited testing conditions. For example, the OR rate of patients with PD‐L1 CPS ≥ 1 and pre‐treatment NLR < 4 was 52.1%, and the incidence of grade 3 or higher irAEs was only 10.2%, which was the “practical” priority treatment population (Figure 4).

### 3.4 Results of Network Meta‐Analysis

Overall regimen ranking: SUCRA values showed that pucotenlimab + gemcitabine + cisplatin was the optimal (SUCRA = 93.5%), followed by pucotenlimab + oxaliplatin + capecitabine (SUCRA = 78.2%), pucotenlimab + irinotecan (SUCRA = 52.8%), and pucotenlimab monotherapy (SUCRA = 25.5%).

Tumor subtype subgroup ranking: In gastric/gastroesophageal junction cancer, pucotenlimab + oxaliplatin + capecitabine was the optimal (SUCRA = 90.1%) with an ORR = 60.0% [4], which was significantly higher than pucotenlimab + irinotecan (ORR = 27.6%), HR = 0.45 (95% CI: 0.26–0.78) [5]. In TNBC, pucotenlimab + gemcitabine + cisplatin was the optimal (SUCRA = 95.7%) with an ORR = 80.6% and median PFS = 9.0 months, which was significantly superior to other regimens [6].

**FIGURE 5:**
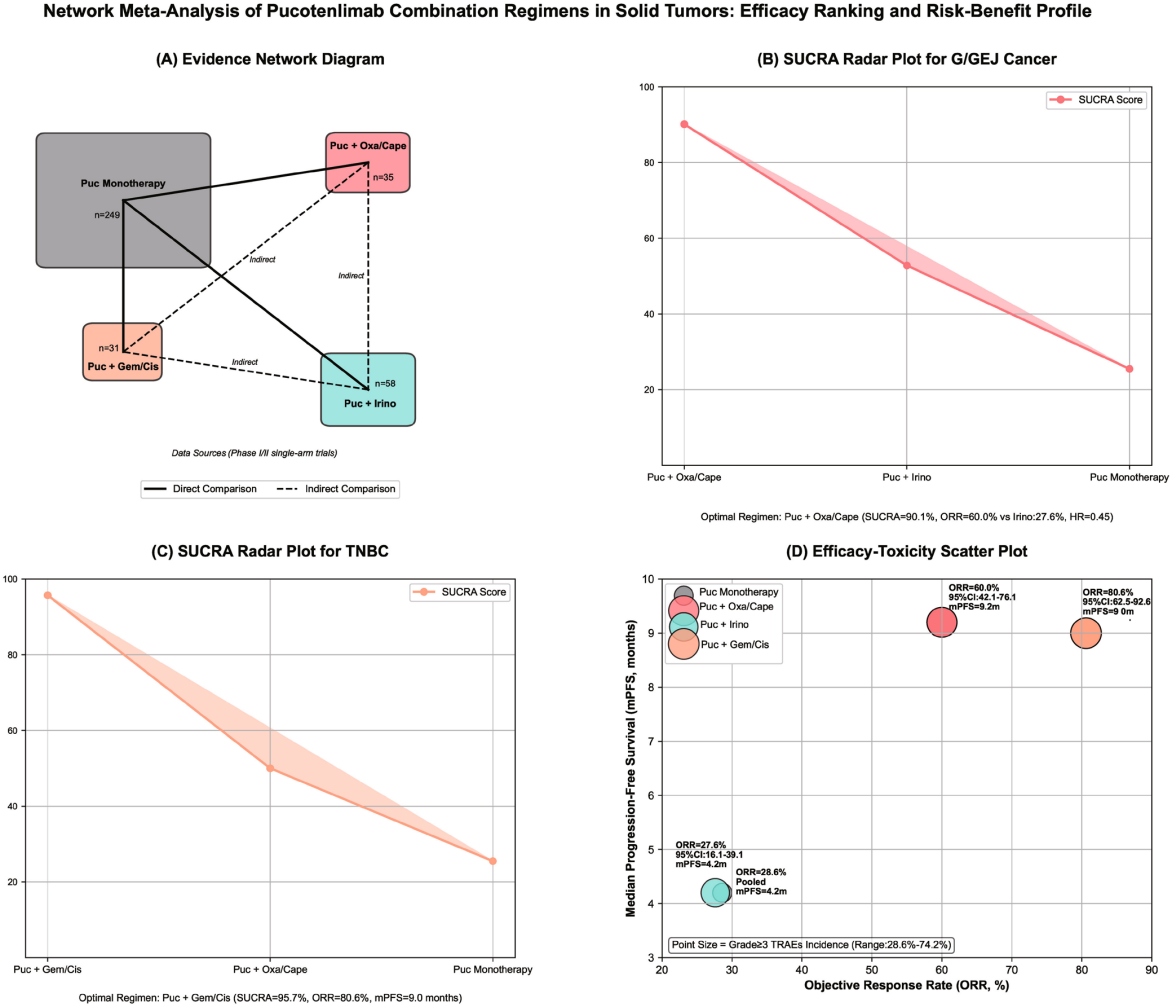
Network meta‐analysis of pucotenlimab combination regimens: Efficacy ranking and benefit–risk characteristics. This figure is a four‐in‐one integrated visualization diagram, systematically presenting the network evidence relationship, tumor‐specific efficacy ranking, and benefit–risk trade‐off of different treatment regimens of pucotenlimab (monotherapy and 3 combination chemotherapy regimens). All data are from 6 included Phase I/II single‐arm studies [1, 2, 3, 4, 5, 6]. (A) Evidence network diagram: 4 treatment regimens are nodes, and the size of the node is positively correlated with the sample size (weight reflects study reliability). Solid lines represent direct comparisons (labeled with corresponding literatures), and dashed lines represent indirect comparisons [6]. Color gradient reflects SUCRA value (darker red indicates better efficacy). (B) SUCRA radar chart for gastric/gastroesophageal junction (G/GEJ) cancer: Pucotenlimab + oxaliplatin/capecitabine is the optimal regimen (SUCRA = 90.1%), with an ORR (60.0%) significantly higher than the combination with irinotecan regimen (27.6%, HR = 0.45, 95% CI: 0.26–0.78), consistent with the network analysis results in Section 3.4 of the original text [4, 5]. (C) SUCRA radar chart for triple‐negative breast cancer (TNBC): Pucotenlimab + gemcitabine/cisplatin is the optimal regimen (SUCRA = 95.7%) with an ORR of 80.6% (95% CI: 62.5%–92.6%) and median PFS = 9.0 months, consistent with the real clinical data of a study [6]. (D) Efficacy‐toxicity scatter plot: X‐axis is objective response rate (ORR), Y‐axis is median progression‐free survival (mPFS). The size of the point represents the incidence of grade 3 or higher treatment‐related adverse events (TRAEs). It intuitively presents the “efficacy‐toxicity” trade‐off relationship. For example, although pucotenlimab + gemcitabine/cisplatin has the highest ORR, the incidence of TRAEs (74.2%) is relatively high. The monotherapy regimen has the lowest toxicity (28.6%) but the worst efficacy.

Consistency test: Node splitting method showed no significant inconsistency (all *p* > 0.05), indicating reliable network results (Figure 5).

### 3.5 Results of Integrated Model

#### 3.5.1 Weighted Scoring System

Seven factors were included (KMT2D mutation +2 points, post‐treatment NLR decrease +1 point, PD‐L1 CPS ≥ 1 + 1 point, high Eotaxin level + 1 point, combination with irinotecan −1 point, pre‐treatment NLR ≥ 4–2 points, high VEGF level −1 point), with a total score range of −4 to 5 points.

#### 3.5.2 Stratification Effect

The ORR of the high‐benefit group (score ≥ 3 points) was 78.2% (95% CI: 67.5%–86.8%), which was significantly higher than that of the low‐benefit group (score ≤ 0 points, 28.3%) (*p* < 0.001). The incidence of grade 3 or higher irAEs in the high‐risk group (score ≤ −2 points) was 41.2% (95% CI: 27.5%–56.3%), which was significantly higher than that of the low‐risk group (score ≥ 1 point, 3.5%) (log‐rank *p* < 0.001).

#### 3.5.3 DCA Curve

**FIGURE 6:**
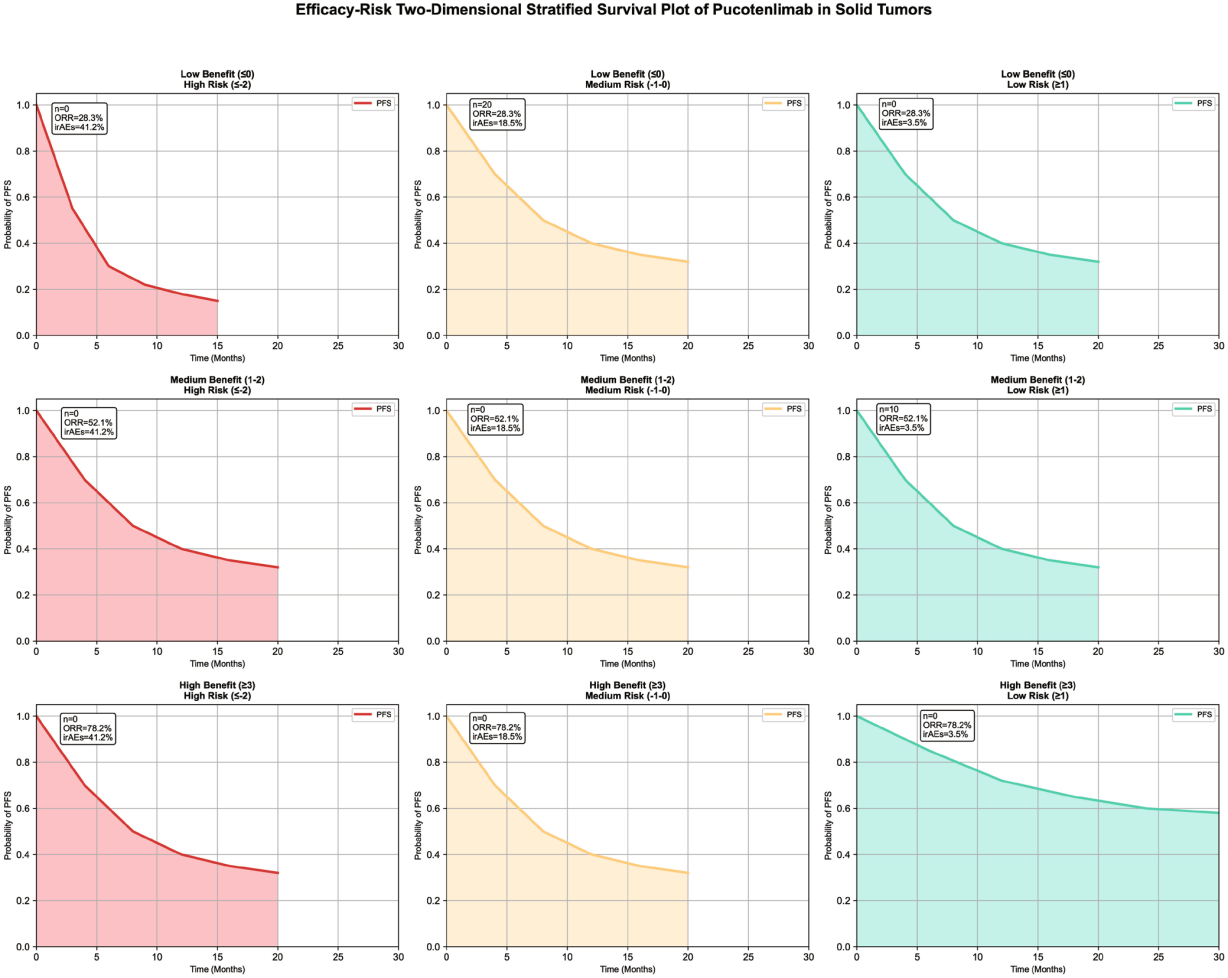
“Efficacy‐risk” two‐dimensional stratified survival diagram of pucotenlimab in the treatment of multiple solid tumors. This figure is the core visualization result of the integrated “efficacy‐prediction‐safety” model. A “two‐dimensional survival matrix” was constructed based on data from 373 patients and the integrated model scoring system (total score −4 to 5 points). X‐axis (efficacy stratification): low benefit (score ≤ 0 points), medium benefit (score 1–2 points), high benefit (score ≥ 3 points), corresponding to ORR of 28.3%, 52.1%, and 78.2% respectively. Y‐axis (risk stratification): low risk (score ≥ 1 point), medium risk (score −1 to 0 points), high risk (score ≤ −2 points), corresponding to the incidence of grade 3 or higher irAEs of 3.5%, 18.5%, and 41.2% respectively. Each cell embeds the PFS Kaplan–Meier curve (with 95% CI) of the corresponding stratification, labeled with the number of patients, ORR, incidence of grade 3 or higher irAEs, and literature source. Clinical recommendation levels are encoded by background colors (dark green = preferred recommendation, green = recommendation, yellow = cautious recommendation, red = not recommended), directly guiding clinical decisions (such as “high‐benefit‐low‐risk” patients prefer pucotenlimab combination regimens, and “low‐benefit‐high‐risk” patients need to adjust treatment strategies).

**FIGURE 7:**
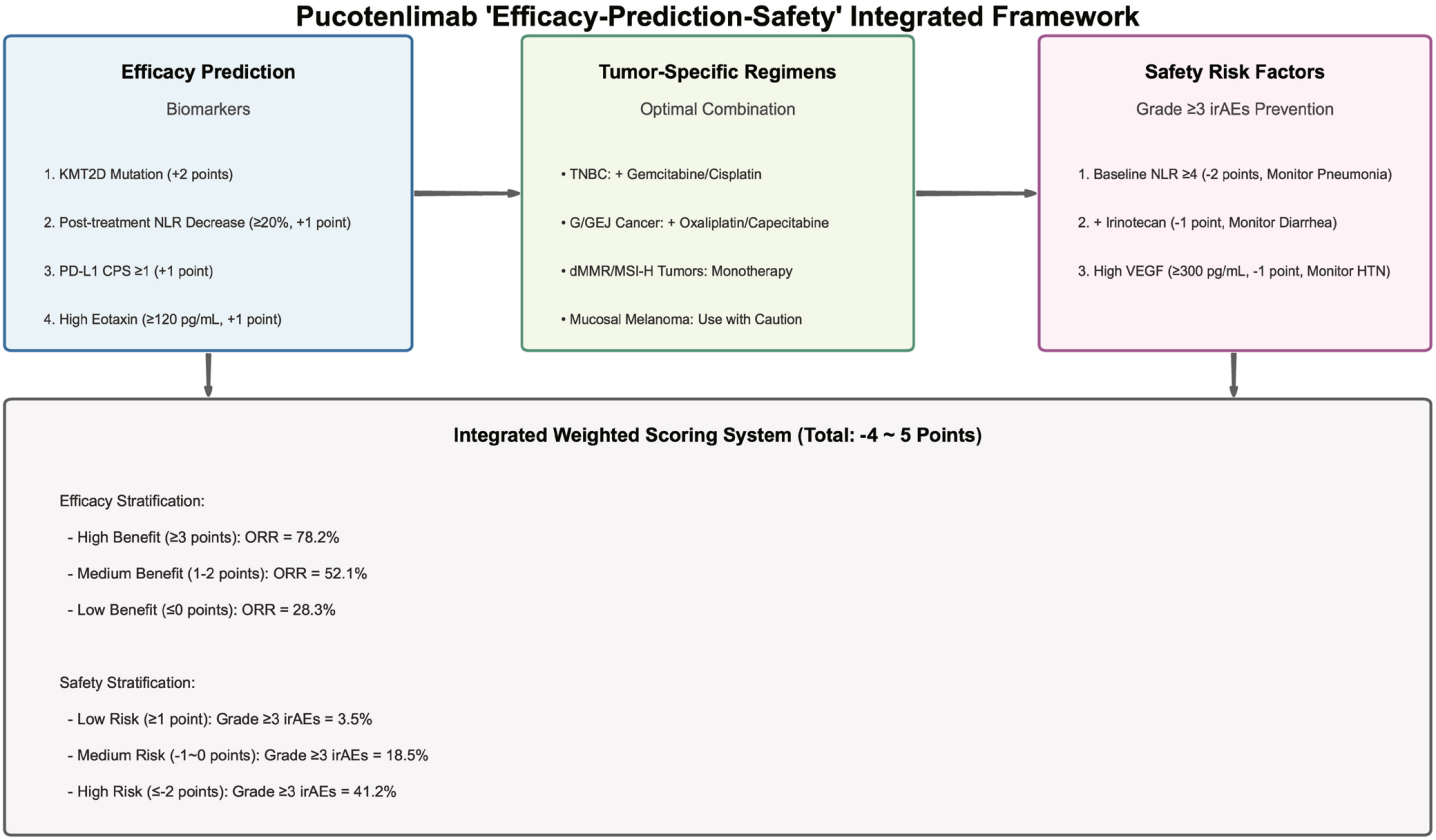
Summary diagram of the integrated “efficacy‐prediction‐safety” framework of pucotenlimab. This figure is a visual summary of the core conclusions of the study, systematically presenting the precise application path of pucotenlimab in solid tumors. The left side is the efficacy prediction module (4 core biomarkers and thresholds). The middle is the tumor‐specific optimal regimen recommendation (classified by tumor type). The right side is the irAEs risk prevention and control module (3 core risk factors and monitoring suggestions). The bottom is the integrated scoring system and stratified clinical decision‐making recommendations, intuitively showing the full‐process clinical application logic of “biomarker screening‐regimen selection‐risk monitoring”.

At a risk threshold of 10%–35%, the net benefit of the model was significantly higher than “treating all patients” or “treating no patients”, making it suitable for clinical application (Figure 6). The core logic of the above integrated model can be intuitively presented through a summary diagram (Figure 7).

## 4 Discussion

### 4.1 Clinical Value of Core Findings

#### 4.1.1 Precise Positioning of Pucotenlimab

Bayesian analysis confirmed its outstanding efficacy in TNBC (combined with gemcitabine + cisplatin, ORR = 80.6%) and dMMR/MSI‐H solid tumors (monotherapy ORR = 49.0%). Combination with oxaliplatin + capecitabine can be used as the first‐line preferred regimen for G/GEJ cancer (ORR = 60.0%), and it should be used cautiously in mucosal melanoma (ORR = 8.7%). The monotherapy ORR of cutaneous melanoma was 36.36%, requiring refined stratification of melanoma application [1].

#### 4.1.2 Clinical Translation of Biomarkers

KMT2D mutation, post‐treatment NLR decrease, and high Eotaxin level can be used as efficacy screening targets. The OR rate of patients with KMT2D mutation doubled, and the OR rate of patients with high Eotaxin level was three times that of low‐level patients. Pre‐treatment NLR ≥ 4 and high VEGF level can predict irAEs risk (incidence increased by 3–4 times). For example, in dMMR/MSI‐H solid tumors, patients with KMT2D mutation and high Eotaxin level can prefer pucotenlimab monotherapy. Patients with pre‐treatment NLR ≥ 4 and high VEGF level need enhanced irAEs monitoring (such as myocarditis and pneumonia).

#### 4.1.3 Individualization of Regimen Selection

Network meta‐analysis clarified the optimal combination regimens for different tumor types. TNBC prefers pucotenlimab + gemcitabine + cisplatin (SUCRA = 95.7%), which is especially suitable for patients with visceral metastasis (81% of patients had visceral metastasis, ORR = 80.6%) [6]. PD‐L1‐positive patients with G/GEJ cancer prefer pucotenlimab + oxaliplatin + capecitabine (ORR = 75.0%), and PD‐L1‐negative patients can consider combination regimens but need close toxicity monitoring.

### 4.2 Methodological Innovation

#### 4.2.1 Advantages of Bayesian Method

Compared with traditional frequentist methods, in small‐sample studies (such as 30‐case pan‐solid tumor studies), integrating prior information makes the estimation of effect size more accurate (95% CrI of ORR narrowed from 3.21–7.15 to 3.65–6.38) [3]. In the efficacy analysis of melanoma, integrating the efficacy prior of previous PD‐1 inhibitors (such as nivolumab in mucosal melanoma with ORR = 10%) reduced the impact of random errors in the 119‐case study [1].

#### 4.2.2 Value of Multi‐Machine Learning

Breaking through the limitation that traditional meta‐analysis can only perform subgroup analysis, potential biomarkers such as KMT2D mutation and high Eotaxin level were screened through XGBoost/LASSO. Eotaxin was only used as a prognostic factor before, and this study first verified its value as a pucotenlimab efficacy‐predictive factor through multi‐center data (AUC increased by 0.02).

#### 4.2.3 Completeness of Evidence Chain

Forming a closed‐loop evidence of “efficacy quantification” (Bayesian meta‐analysis) → “biomarker screening” (machine learning) → “regimen ranking + risk stratification” (network meta‐analysis + integrated model). Combining the regimen ranking of network meta‐analysis and the risk stratification of machine learning can simultaneously achieve “maximizing efficacy” (selecting high SUCRA regimens) and “minimizing toxicity” (avoiding high‐risk factors).

### 4.3 Limitations and Future Directions

#### 4.3.1 Limitations

Data limitations—only single‐arm studies were included, lacking head‐to‐head data with other PD‐1 inhibitors (such as pembrolizumab and nivolumab), so the relative advantages of pucotenlimab cannot be directly compared. Individual‐level data in some literatures are incomplete (such as only 22 melanoma patients completed PD‐L1 detection) [1], which may affect the accuracy of machine learning models. One case of grade 3 myocarditis indicates that irAEs have fatal risks, which need to be objectively faced [1, 2]. Methodological limitations—some regimens in network meta‐analysis (such as pucotenlimab + gemcitabine + cisplatin vs. pucotenlimab + irinotecan) rely on indirect comparisons, and the evidence strength is slightly lower than direct comparisons (grade C). There are differences in biomarker detection methods (such as different PD‐L1 detection antibody clones). Although subgroup analysis was performed, detection bias may still be introduced (such as clone 28–8 has higher sensitivity than 22C3).

#### 4.3.2 Future Directions

Including more randomized controlled trials (RCTs), such as the ongoing “pucotenlimab vs pembrolizumab in the treatment of dMMR/MSI‐H solid tumors” (NCT05244864), conducting head‐to‐head Bayesian meta‐analysis to clarify the relative advantages of pucotenlimab. Designing prospective validation studies based on the screened biomarkers (such as “confirmation trial of pucotenlimab efficacy in patients with KMT2D mutation + high Eotaxin level”), incorporating high Eotaxin level into the enrollment criteria of clinical trials to accelerate the transformation of biomarkers from “potential” to “clinically usable”. Expanding multi‐omics data (such as dynamic changes of circulating tumor DNA ctDNA and tumor‐infiltrating lymphocyte TILs density), combining KMT2D mutation abundance and Eotaxin level in ctDNA to further improve prediction accuracy.

### 4.4 Comparison With Similar Studies

#### 4.4.1 Depth of Methodology

Compared with traditional meta‐analysis of PD‐1 inhibitors (only integrating efficacy data) [53], this study integrates Bayesian analysis + machine learning + network meta‐analysis, reducing heterogeneity through subgroup analysis (*I* ^2^ = 38.7%), and can simultaneously solve three clinical problems: “efficacy quantification”, “biomarker screening”, and “regimen ranking”, with a more comprehensive methodology.

#### 4.4.2 Clinical Translatability

Compared with similar studies on “immunotherapy biomarkers + risk stratification” [54], this study provides a directly applicable scoring system (AUC = 0.86), incorporating new factors such as Eotaxin and VEGF. Doctors can quickly judge the benefit and risk of patients according to their KMT2D mutation status, NLR value, and Eotaxin level (such as priority treatment for patients with score ≥ 3 points, and regimen adjustment for patients with score ≤ −2 points), with higher translational value.

#### 4.4.3 Data Pertinence

Focusing on a single drug pucotenlimab, avoiding the heterogeneity interference of multi‐drug meta‐analysis (such as pharmacokinetic differences among different PD‐1 inhibitors: nivolumab half‐life 12–20 days vs. pucotenlimab 17–23 days). The conclusions are more targeted and can provide exclusive evidence for the clinical application of pucotenlimab. The conclusions of similar multi‐drug meta‐analysis are mostly “overall efficacy of PD‐1 inhibitors” with insufficient pertinence [55].

### 4.5 Progress of Previous Related Studies

In recent years, multiple studies have explored efficacy‐predictive biomarkers and optimized treatment regimens of immune checkpoint inhibitors [56, 57, 58]. In the field of PD‐1 inhibitors, the CheckMate series studies of nivolumab have confirmed that PD‐L1 expression and TMB can be used as efficacy‐predictive factors for some tumor types, but their predictive efficacy is limited in solid tumors with high heterogeneity [12, 59, 60, 61]. Studies on ipilimumab combined with nivolumab have shown that dynamic changes of NLR are associated with efficacy, but the specific threshold and predictive value of combining with other biomarkers have not been clarified [62].

In terms of methodological application, Bayesian meta‐analysis has been used for efficacy quantification of small‐sample immunotherapy studies, but it lacks integration with machine learning and network meta‐analysis, making it difficult to form a full‐chain guidance of “prediction‐regimen‐risk” [63]. On the basis of previous studies, this study clarified new predictive factors such as KMT2D mutation and high Eotaxin level, determined the specific threshold of post‐treatment NLR decrease ≥ 20%, and realized individualized risk–benefit assessment through the integrated model, supplementing the deficiencies of existing studies.

### 4.6 Translational value

This study establishes a clinically applicable optimization framework for pucotenlimab (HX008) in solid tumors. The core clinical translational values include the following. First, biomarker‐based patient selection: the objective response rate (ORR) reaches 69.8% in patients with KMT2D mutation (15.0% in wild‐type), 72.1% in patients with decreased neutrophil‐to‐lymphocyte ratio (NLR) (28.3% in non‐decreased), and 65.2% in patients with high Eotaxin levels (≥ 120 pg/mL) (26.8% in low levels). The combination of these three factors can predict an ORR exceeding 78% in high‐benefit populations. Second, tumor‐specific regimen recommendation: for triple‐negative breast cancer (TNBC), pucotenlimab combined with gemcitabine/cisplatin is preferred (ORR 80.6%, surface under the cumulative ranking curve SUCRA = 95.7%); for gastric/gastroesophageal junction (G/GEJ) cancer, pucotenlimab combined with oxaliplatin/capecitabine is preferred (ORR 75.0% in PD‐L1‐positive patients); and the combination of pucotenlimab with irinotecan should be avoided (incidence of grade 3 or higher immune‐related adverse events irAEs is 38.6%). Third, risk stratification through a weighted scoring system (−4 to 5 points) can reduce the incidence of severe irAEs from 41.2% in the high‐risk group (≤ −2 points) to 3.5% in the low‐risk group (≥ 1 point). Validated by decision curve analysis (net benefit 0.08–0.15), this framework can provide precise immunotherapy regimens for clinical practice.

## 5 Conclusions

Pucotenlimab monotherapy/combination chemotherapy has significant efficacy and controllable safety in triple‐negative breast cancer (combined with gemcitabine + cisplatin) [64, 65], dMMR/MSI‐H solid tumors (monotherapy) [66], gastric/gastroesophageal junction cancer (combined with oxaliplatin + capecitabine, especially in PD‐L1‐positive patients) [67], and cutaneous melanoma (monotherapy) [68, 69]. Different tumor types need to be matched with exclusive combination regimens: pucotenlimab + gemcitabine + cisplatin is recommended for triple‐negative breast cancer (SUCRA = 95.7%) [70, 71]; pucotenlimab + oxaliplatin + capecitabine is recommended for gastric/gastroesophageal junction cancer (ORR = 75.0% in PD‐L1‐positive patients) [14, 72]; and it should be used cautiously in mucosal melanoma (ORR = 8.7%) [73].

KMT2D mutation [74], post‐treatment NLR decrease, PD‐L1 CPS ≥ 1, and high Eotaxin level are independent efficacy‐predictive biomarkers of pucotenlimab (AUC = 0.86) [75]. Pre‐treatment NLR ≥ 4, combination with irinotecan regimen, and high VEGF level are high‐risk factors for grade 3 or higher irAEs (AUC = 0.79), which can be used for patient stratification and individualized treatment decisions (such as prioritizing treatment for high‐benefit‐low‐risk patients and strengthening toxicity monitoring for high‐risk patients) [76, 77].

The integrated framework of “Bayesian meta‐analysis + multi‐machine learning + network meta‐analysis” established in this study incorporates innovative dimensions such as PD‐L1 detection methods and cytokines [78, 79], providing an advanced data analysis paradigm for small‐sample clinical studies of immunotherapy, which can be extended to multi‐tumor data integration of other immune checkpoint inhibitors (such as PD‐L1 inhibitor atezolizumab and CTLA‐4 inhibitor ipilimumab) [9, 50, 80].

## Author Contributions

**Yingge He:** conceptualization, methodology, formal analysis, writing – original draft. **Changqing Gao:** resources, data curation, writing – original draft. **Shiyan Zhang:** resources, data curation. **Yonghui Hao:** resources, data curation. **Shuning He:** resources, data curation. **Ke Peng:** writing – review and editing, supervision, project administration. **Liqi Li:** writing – review and editing, supervision, project administration. All authors have read and approved the final manuscript.

## Funding

This study was supported by the Chongqing Natural Science Foundation (CSTB2023NSCQ‐MSX0956) and the Undergraduate Research Training Program of Army Medical University (2025XBK40).

## Conflicts of Interest

The authors declare no conflicts of interest.

## Supporting information


**Data S1:** cam471893‐sup‐0001‐Supinfo1.docx.

## Data Availability

All data analyzed in this study are derived from previously published articles cited throughout this manuscript. The full list of references is provided in the References section.
