# Supplementary material for: Integration Analysis of Bayesian and Machine Learning for Heterogeneity, Biomarkers, and Optimal Combination Regimens of Pucotenlimab in Solid Tumors
Source: Cancer Med. 2026 Apr 27;15(5):e71893. doi: 10.1002/cam4.71893 (PMC13117215; doi:10.1002/cam4.71893)
Supplement: Supplementary file 1 — Data S1: cam471893‐sup‐0001‐Supinfo1.docx. [file CAM4-15-e71893-s001.docx]

**Identification of studies via databases and registers**

Records removed *before screening*:

Duplicate records removed

(n = 14)

Records marked as ineligible by automation tools (n = 0)

Records removed for other reasons (n = 0)

Records identified from*:

Databases (n =31)

Registers (n = 0)

**Identification**

Records screened

(n =17)

Records excluded

(n = 7)

Reports sought for retrieval

(n = 10)

Reports not retrieved

(n = 0)

**Screening**

Reports excluded:

Incomplete data

(n = 3)

Study design mismatch

(n = 1)

Reports assessed for eligibility

(n = 10)

Studies included in review

(n = 6)

Reports of included studies

(n = 0)

**Included**

*Consider, if feasible to do so, reporting the number of records identified from each database or register searched (rather than the total number across all databases/registers).

**If automation tools were used, indicate how many records were excluded by a human and how many were excluded by automation tools.

Source: Page MJ, et al. BMJ 2021;372:n71. doi: 10.1136/bmj.n71.

This work is licensed under CC BY 4.0. To view a copy of this license, visit <https://creativecommons.org/licenses/by/4.0/>
